# Endemic Infection of the Amphibian Chytrid Fungus in a Frog Community Post-Decline

**DOI:** 10.1371/journal.pbio.0020351

**Published:** 2004-10-05

**Authors:** Richard W. R Retallick, Hamish McCallum, Rick Speare

**Affiliations:** **1**School of Tropical Biology, James Cook UniversityTownsville, QueenslandAustralia; **2**Department of Zoology and Entomology, University of QueenslandSt Lucia, QueenslandAustralia; **3**School of Life Sciences—Biology, Arizona State UniversityTempe, ArizonaUnited States of America; **4**Amphibian Diseases Group, School of Public Health and Tropical Medicine, James Cook UniversityTownsville, QueenslandAustralia

## Abstract

The chytrid fungus Batrachochytrium dendrobatidis has been implicated in the decline and extinction of numerous frog species worldwide. In Queensland, Australia, it has been proposed as the cause of the decline or apparent extinction of at least 14 high-elevation rainforest frog species. One of these, *Taudactylus eungellensis,* disappeared from rainforest streams in Eungella National Park in 1985–1986, but a few remnant populations were subsequently discovered. Here, we report the analysis of B. dendrobatidis infections in toe tips of T. eungellensis and sympatric species collected in a mark-recapture study between 1994 and 1998. This longitudinal study of the fungus in individually marked frogs sheds new light on the effect of this threatening infectious process in field, as distinct from laboratory, conditions. We found a seasonal peak of infection in the cooler months, with no evidence of interannual variation. The overall prevalence of infection was 18% in T. eungellensis and 28% in *Litoria wilcoxii/jungguy,* a sympatric frog that appeared not to decline in 1985–1986. No infection was found in any of the other sympatric species. Most importantly, we found no consistent evidence of lower survival in T. eungellensis that were infected at the time of first capture, compared with uninfected individuals. These results refute the hypothesis that remnant populations of T. eungellensis recovered after a B. dendrobatidis epidemic because the pathogen had disappeared. They show that populations of T. eungellensis now persist with stable, endemic infections of B. dendrobatidis.

## Abstract 2

Recapture experiments provide evidence that some amphibian species can now persist with infections of the pathogenic chytrid fungus and suggests, for example, that frogs and fungus might be coevolving

## Introduction

Increasingly, the amphibian chytrid fungus *(Batrachochytrium dendrobatidis)* has been implicated as a major contributor to global catastrophic declines in frog populations (Berger et al. 1998; Daszak et al. 1999, 2003). It has been found on frogs in areas where catastrophic declines were reported, it has been shown in the laboratory to be highly pathogenic to some species, and there is pathological evidence to link this fungal parasite to host mortality (Berger et al. 1998). The pathogen may therefore be capable of producing the extremely high mortality observed during declines. However, little information is available on the impact of the fungus on individuals in the field, rather than the laboratory. Furthermore, little has been published on the prevalence of infection among frog populations as a whole, as distinct from the prevalence among morbid frogs only. In addition to data on the prevalence of the pathogen among morbid animals, information on the prevalence of a putative pathogen in the population in general is important to determine the potential effect of the pathogen on the host population (McCallum and Dobson 1995).

In Queensland, Australia, there have been extinctions or major declines of at least 14 frog species in undisturbed, high-elevation rainforest streams, commencing in 1979–1981 in the Conondale and Blackall Ranges (26°50′ S, 152°41′ E), followed in 1985–1986 in the Eungella region of the Clarke Range (21°07′ S, 148°29′ E), and, in 1990–1995, in the Wet Tropics bioregion (17°22′ S, 145°49′ E) (Laurance et al. 1996; McDonald and Alford 1999). Laurance et al. (1996, 1997) suggested that an epidemic disease was responsible for all these declines, without proposing an agent. Despite the presence of B. dendrobatidis in ill and dead frogs collected from the Big Tableland in the Wet Tropics in 1993, it was not recognised as a pathogenic organism until 1998 (Berger et al. 1998) and was described as a new species of fungus in 1999 (Longcore et al., 1999). B. dendrobatidis has been suggested as the causative agent of many of the east coast Australian declines (Berger et al. 1999a), although only the decline at Big Tableland (McDonald and Alford 1999) had direct evidence of an association with the presence of B. dendrobatidis.

**Figure 1:**
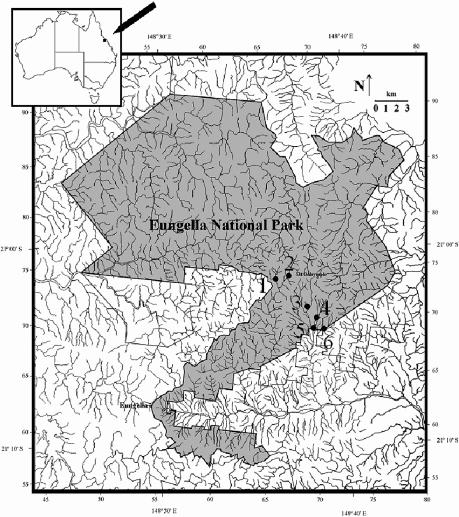
Location of Study Sites 1, Mount David Creek; 2, Mount William Creek; 3, Dooloomai Falls; 4, Rawson Creek; 5, Picnic Ground Creek; and 6, Tree Fern Creek.

In this paper, we report the retrospective analysis of B. dendrobatidis infection on toe tips collected between 1994 and 1998 from six species of frogs at Eungella National Park in east-central Queensland, Australia (Figure 1). Declines at this location were particularly catastrophic. Between 1985 and 1986, the Eungella Gastric-Brooding Frog *(Rheobatrachus vitellinus)* disappeared suddenly from relatively undisturbed rainforest streams, and it is now considered to be extinct (McDonald 1990; Campbell 1999; Department of Environment and Heritage 2003). During the same period, the Eungella Torrent Frog *(Taudactylus eungellensis)* also disappeared, but it was later found to have persisted in a few small populations (McDonald 1990; Couper 1992; McNellie and Hero 1994; Retallick et al. 1997), which are the subject of this paper. A suite of other species that coexisted with R. vitellinus and T. eungellensis showed no evidence of decline. Unfortunately, the extent of infection with B. dendrobatidis in the frog community at Eungella during the period of decline is unknown. The first record of B. dendrobatidis at Eungella was from a moribund frog collected in 1995 (Berger et al. 1999a).

## Results

We detected B. dendrobatidis on 71 (15.0%) of the 474 toes assessed. Four species showed no sign of infection, while we detected infections on 58 (18.4%) of 316 toes of T. eungellensis and 13 (34%) of 47 toes of frogs identified at the time as Litoria lesueuri (Table 1). Recently, the taxonomy of L. lesueuri has been revised (Donellan and Mahoney, 2004), with North Queensland members of the complex being either L. jungguy or *L. wilcoxii.* From the reported distributions of these species, our *“L. lesueuri”* may have been either one of the species or even hybrids (Donnellan and Mahoney, 2004; Michael Mahoney, personal communication) These species can be distinguished only with genetic information. We therefore describe them as *L. wilcoxii/jungguy* for the remainder of this paper.

**Table 1:**
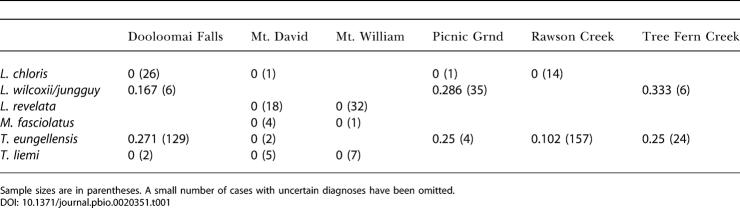
Prevalence of Infection of B. dendrobatidis in All Frog Toes Examined

Sample sizes are in parentheses. A small number of cases with uncertain diagnoses have been omitted

Using a logistic model with season (summer, autumn, winter, and spring), species, and site as predictor variables, we found significant effects of each variable on infection, corrected for the effects of the other variables (for season, the change in deviance [Δdev] = 15.25, df = 3, *p* = 0.0061; for site, Δdev = 13.56, df = 5, *p* = 0.02; for species, Δdev = 17.32, df = 5, *p* = 0.004). The most parsimonious model (i.e., one that minimizes the Akaike Information Criterion [AIC]) included each of these predictors but no interaction terms.

Further analysis was concentrated on the two species on which B. dendrobatidis was detected, namely T. eungellensis and *L. wilcoxii/jungguy.*

### Infection in T. eungellensis

The proportions of frogs that were infected in the three largest populations (at Rawson Creek, Dooloomai Falls, and Tree Fern Creek [Figure 1]) differed significantly among those sites (Δdev = 12.84, df = 2, *p* = 0.001). A significantly smaller proportion was infected at Rawson Creek (10.5%) than at Dooloomai Falls (26.7%) or Tree Fern Creek (25.0%). There was a marginally significant difference overall in the infection levels among males, females, and subadults (Δdev = 5.9, df = 2, *p* = 0.052). However, when site was included in the model, any suggestion of a difference in infection level between males, females, or subadults disappeared. When we separated the main T. eungellensis populations, it was apparent that the difference in prevalence between those categories was influenced by the population at Tree Fern Creek, where 46% of males (*n* = 13) and no females or subadults were infected (Table 2). When we grouped all sites and times, the estimated overall prevalence of infection was 18.1%.

**Table 2:**
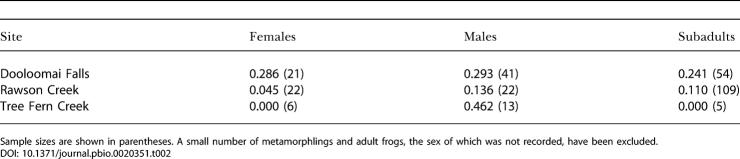
Prevalence of Infection of B. dendrobatidis in T. eungellensis by Age/Sex Class and Site, Pooled over Sampling Times

Sample sizes are shown in parentheses. A small number of metamorphlings and adult frogs, the sex of which was not recorded, have been excluded

**Figure 2:**
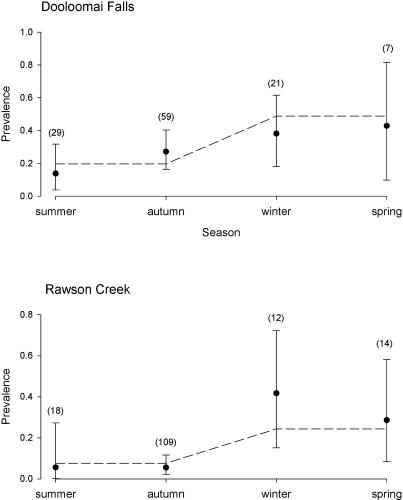
Seasonal Patterns of Prevalence of B. dendrobatidis in T. eungellensis Solid circles show the observed prevalence, with binomial 95% confidence limits, in frogs pooled over years and age/sex class. The dashed line shows the prevalence predicted from the best-fitting logistic model. Numbers in brackets above each error bar are the sample sizes.

At these three sites, levels of infection among T. eungellensis varied significantly among seasons (Δdev = 14.605, df = 3, *p* = 0.002), but not among years (Δdev = 3.433, df = 3, *p* = 0.26). Further, there was no evidence that the pattern of seasonal variation in infection changed among years (Δdev = 5.561, df = 6, *p* = 0.49). Infection was most prevalent (37.8%) during the winter months (1 June to 31 August) and least prevalent (11.3%) during the summer months (1 December to 28/29 February). Comparing the two sites with the largest sample sizes, Dooloomai Falls and Rawson Creek, there was no evidence that they had differing seasonal patterns of infection (Δdev = 5.32, df = 3, *p* = 0.150), although the level of infection overall was much higher at Dooloomai Falls (log odds ratio = 1.1612, standard error [se] = 0.3586, *p* = 0.0012). Infection levels were much higher in winter and spring combined than in summer and autumn (log odds ratio = 1.360, se = 0.3589, *p* = 0.00015), and there was no evidence of infection levels differing between winter and spring or between summer and autumn (Δdev = 1.107, df = 2, *p* = 0.5). The seasonal changes in infection at the two sites are shown in Figure 2.

### Infection in *L. wilcoxii/jungguy*

B. dendrobatidis was detected in 13 of the 47 *L. wilcoxii/jungguy* examined, giving an estimated prevalence of 27.7%, with a 95% confidence interval ranging from 15.6% to 42.6%. There was no evidence that prevalence of infection differed among males, females, and subadults (Δdev = 2.32, df = 2, *p* = 0.31); among sites (Δdev = 0.175, df = 2, *p* = 0.91); or among seasons (Δdev = 1.44, df = 1, *p* = 0.7) (each of the above Δdev terms is corrected for the other terms in the model).

Comparing the prevalence of infection between *L. wilcoxii/jungguy* and T. eungellensis was hampered by the fact that prevalence of infection on T. eungellensis differed between sites, and that the sampled populations of the two frog species had largely disjunct distributions. When the data were pooled over all sites, there was insufficient evidence to reject a null hypothesis of equal prevalence in the two species, whether the effects of season were allowed for (Δdev = 0.15, df = 1, *p* = 0.7) or not (Δdev = 2.09, df = 1, *p* = 0.15).

### Influence of Infection on Recapture and Survival

Table 3 shows the numbers of *L. wilcoxii/jungguy* and T. eungellensis recaptured at any stage later in the study, grouped by their infection status at first capture. A logistic model with recapture as the response and species and infection status as predictors produced no evidence that the effect of B. dendrobatidis infection on recapture probability differed between the species (Δdev = 0.002, df = 1, *p* = 0.96). In both species, the probability of recapture was significantly lower for infected frogs than for uninfected frogs (Δdev = 5.34, df = 1, *p* = 0.02; log odds ratio = –0.6464, se = 0.2856), correcting for the substantially higher overall recapture rate of T. eungellensis relative to *L. wilcoxii/jungguy*.

**Table 3:**
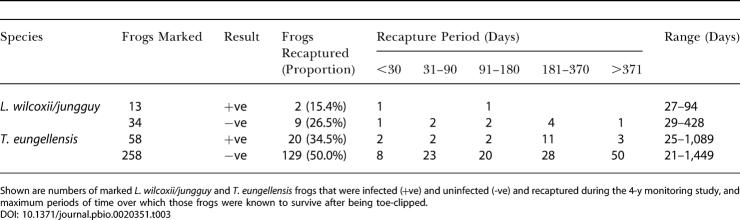
Recapture and Survival of Infected and Uninfected Frogs

Shown are numbers of marked *L. wilcoxii/jungguy* and T. eungellensis frogs that were infected (+ve) and uninfected (-ve) and recaptured during the 4-y monitoring study, and maximum periods of time over which those frogs were known to survive after being toe-clipped

This simple analysis suggests that B. dendrobatidis may affect survival, but confounds the probability of recapturing a frog that is present at a site with the probability that a frog is still present at the site. The more sophisticated analysis that follows separates these two components, although it cannot distinguish between death and permanent emigration from the site. For brevity, we refer to continued presence at the site as “survival.”

**Figure 3:**
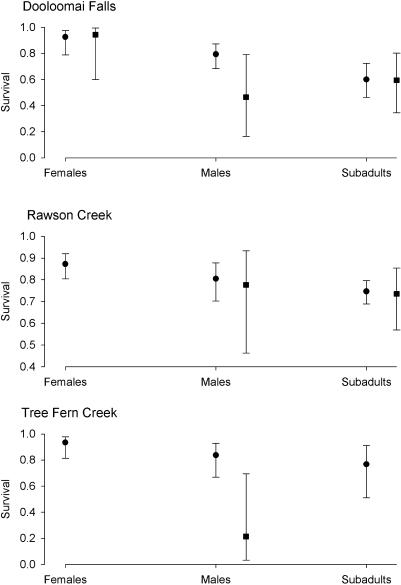
Estimated Quarterly Survival of Age/Sex Classes of Taudactylus eungellensis at Three Sites Survival for uninfected frogs is shown with circles, and survival for infected frogs is shown with squares; 95% confidence limits around each point are also shown. Where there is no survival estimate for infected frogs, there were insufficient data to estimate the parameter. Sample sizes are given in Table 2.

Investigating mark–recapture data for both Rawson Creek and Dooloomai Falls, we found that the best model (on the basis of minimizing the AIC) for which there were sufficient data to estimate almost all parameters had the probability of recapture varying with time but not group (i.e., infected and uninfected males, females, and subadults), and the survival probability differing between the groups but constant through time. However, further analysis in both cases showed that there was no evidence that survival differed between infected and uninfected frogs: At both sites, the group effect was due to survival being highest in females, intermediate in males, and lowest in subadults (Figure 3; Table 4). In the case of Tree Fern Creek, which had a smaller total sample size than the other two sites, and in which only males were infected, the best model had recapture probabilities constant with both time and group, and survival varying with group. At this site, there was some evidence that infected males had lower survival than uninfected males (Figure 3; Table 4).

**Table 4:**
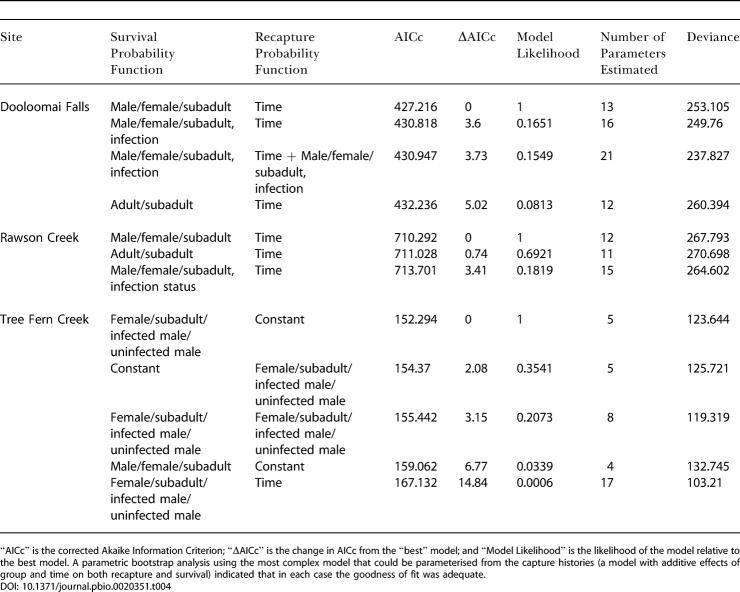
Summary of Results of Mark-Recapture Modelling on T. eungellensis

“AICc” is the corrected Akaike Information Criterion; “ΔAICc” is the change in AICc from the “best” model; and “Model Likelihood” is the likelihood of the model relative to the best model. A parametric bootstrap analysis using the most complex model that could be parameterised from the capture histories (a model with additive effects of group and time on both recapture and survival) indicated that in each case the goodness of fit was adequate

## Discussion

Our results show unequivocally that remnant populations of *T. eungellensis,* a rainforest frog that almost disappeared as a result of major die-offs in the Eungella area in the mid 1980s, now persist with stable infections of *B. dendrobatidis.* This does not imply that this pathogen cannot have been responsible for the decline. One hypothesis that can be discarded, however, is that B. dendrobatidis drove frog populations to low levels, consequently became locally extinct itself, and that frog populations subsequently recovered or stabilised in the absence of the pathogen. Two other hypotheses are consistent with our observed results.

It may be that B. dendrobatidis was not responsible for the initial decline of T. eungellensis populations. These populations declined more than a decade before this chytrid was formally identified, and the declines occurred over a very short period (McDonald 1990). Because of the rapidity of the declines, no samples were collected during the period of declines, so it is not possible to examine for the presence of *B. dendrobatidis.* Although it is not possible to eliminate all environmental factors as being responsible for the decline of T. eungellensis and the disappearance of R. vitellinus from Eungella in 1985, the failure to detect substantial climatic anomalies in the area at that time (McDonald 1990) means that this explanation is unlikely to account for the sudden declines.

Another possibility consistent with our results is that B. dendrobatidis was a pathogen novel to the ecosystem in 1985, and that it was indeed responsible for the declines. Available molecular evidence (Morehouse et al. 2003), although not conclusive, is consistent with the hypothesis that B. dendrobatidis is a recently emerged disease agent. Populations of T. eungellensis may have recovered or stabilised following evolution of resistance to the pathogen in the frogs, or evolution of less-pathogenic strains of the fungus. Rapid coevolutionary changes in both host and pathogen following the introduction of a novel pathogen are to be expected, with the best-known example being the coevolution of myxomatosis and its rabbit hosts within a few years of the introduction of the virus to Australia in 1953 (May and Anderson 1983; Fenner and Fantini 1999). Our study provides no direct evidence for such coevolution in frogs and *B. dendrobatidis,* but it identifies this as an important area for future research. If it could be shown that susceptible frog populations were able to develop resistance to the amphibian chytrid, then captive breeding and artificial selection for resistance would provide an avenue for management of this threat to critically endangered species.

It is intriguing that we found similar seasonal fluctuations in infection levels within each year, but no evidence of variation among the 4 y of the study. This suggests strongly that B. dendrobatidis has become endemic and relatively stable in prevalence in these populations. Given that frog numbers and diversity remained broadly similar over this period, it suggests that some form of host–pathogen equilibrium has become established, in contrast to the epizootic that may have occurred 10 y previously.

There are some obvious methodological limitations in our study that we must acknowledge. Infection status was determined by histological examination of toe tips at the time of first capture. Toe tips are often examined for B. dendrobatidis because the feet of frogs are a particularly favoured body location for infection by the fungus (Berger 2001). However, in light infections, B. dendrobatidis can occur in microscopic clusters, which can potentially be missed in histological sections. Hence, a proportion of frogs found to be negative for B. dendrobatidis using histology at first capture may actually have been infected. This possibility of false negatives (Berger 2001; Berger et al. 2002) suggests that the infection prevalences found in this study may underestimate the true prevalence of B. dendrobatidis within these frog populations. For individuals classed as infected at the time of first capture, and that subsequently survived for extended periods, it is not known whether their infection persisted or was cleared after first capture. There is evidence that some frog species can clear B. dendrobatidis infection: 50% of experimentally infected Mixophyes fasciolatus held at 27 °C with confirmed infection subsequently cleared the infection (Berger 2001; Berger et al. 2004). The prevalences we report are therefore estimated from the prevalence of infection in frogs captured for the first time in the period under consideration.

In this study, infection levels of B. dendrobatidis were significantly higher during winter and spring than during summer and autumn. A survey of ill and dead frogs from eastern Australia from 1995 to 1999 showed a similar seasonal prevalence of chytridiomycosis (Berger et al. 2004). In the laboratory, the growth of B. dendrobatidis has been shown to peak at about 23 °C, with death or arrested growth occurring in vitro at temperatures above 30 °C (Longcore et al. 1999; Johnson et al. 2003; Piotrowski et al. 2004). Further, infection in some frog species can be cleared in the laboratory by exposing them to temperatures in excess of 37 °C (Woodhams et al. 2003). At Eungella, 23 °C is a typical daytime maximum temperature for winter, with water and air temperatures along streams generally remaining at or below 23 °C for the entire 24-h daylength period. In summer, however, daytime air temperatures along Eungella's streams regularly exceed 23 °C, and at sites such as Rawson Creek can reach as high as 37 °C (R. Retallick, unpublished data).

The different infection levels among sites for T. eungellensis reported in Table 2 may be correlated with the degree of sunlight and warmth that reaches the streams. The streams at Dooloomai Falls and Tree Fern Creek are well shaded and remain relatively cool and damp for much of the year. At those sites, the average canopy gap above the centre of the streams (a coarse measure of how much sunlight reaches the stream) are 0.35 m and 1.90 m respectively (R. Retallick, unpublished data). Rawson Creek is considerably wider (average canopy gap = 5.10 m) and thus receives more sunlight. With access to sunny microhabitats, where surface temperatures can exceed ambient air temperatures, frogs at “warmer” streams such as Rawson Creek may be less subject to infection at any time of the year, or may be able to reduce infection through thermoregulation. The idea that frogs may avoid, control, or eliminate infection by differential use of their environment warrants considerable and immediate attention. Such a process may prove to be critical to the relationship between B. dendrobatidis and frog populations in the wild.

Our mark–recapture analysis did not find consistent evidence that T. eungellensis infected with B. dendrobatidis at the time of first capture had a lower survival rate than uninfected *T. eungellensis.* Failure to reject the null hypothesis of no effect of infection on survival cannot, of course, be used as evidence in favor of the null hypothesis, and it is worth noting that the point estimate of survival for infected males was lower than that for uninfected males at each site. Only at Tree Fern Creek, however, did the best-supported model include an infection term. There was also evidence that infection influenced the proportion of frogs that were recaptured (Table 3). Together with the observation that some infected T. eungellensis survived for extended periods (a maximum of 1,089 d), our results show that infection with B. dendrobatidis did not inevitably lead to rapid death in *T. eungellensis.*

Both epidemiological theory and observations suggest that where a pathogen drives a host species to extinction, there is likely to be a reservoir host within which the pathogen has a reduced effect and is therefore maintained at a higher prevalence (McCallum and Dobson 1995, 2002). *L. wilcoxii/jungguy* appears not to have declined at the same time as T. eungellensis and R. vitellinus and, in our data, it has a high prevalence of infection. It therefore is a candidate reservoir host. Whether the prevalence of B. dendrobatidis differs between the sibling species L. wilcoxii and L. jungguy at sites where they are sympatric would be an interesting question, but cannot be answered from our study. The L. lesueuri complex has a widespread distribution throughout streams on the east coast of Australia (Barker et al. 1995; Donellan and Mahony 2004) and could therefore play a substantial role in the maintenance and spread of chytrid infection throughout that region. If a species is acting as a reservoir, the prevalence of infection in that species should be higher than in species that are declining as a result of infection (McCallum and Dobson 1995). The prevalence of infection we observed in *L. wilcoxii/jungguy* exceeded that observed in *T. eungellensis,* but we do not have clear evidence that this represents a higher prevalence in the populations as a whole. It is also possible that B. dendrobatidis exists as a saprophyte in the environment independent of amphibians (Longcore et al. 1999; Johnson and Speare 2003), or may use tadpoles (which seem relatively little affected by the pathogen) as a reservoir (Daszak et al. 1999).

We did not detect infection in any of the 50 individuals of Litoria revelata or the 42 individuals of L. chloris we examined, which demonstrates that those species had a lower prevalence of chytridiomycosis than T. eungellensis and *L. wilcoxii/jungguy*. We did not record any infected frogs of any species from the two sites at which L. revelata was collected. *Litoria chloris,* however, has been shown to carry infection in other areas (Speare and Berger 2004) and in laboratory experiments (Woodhams et al. 2003). Sample sizes in our study for these other frog species may be too small to draw any reliable conclusions about whether they become infected with B. dendrobatidis in the wild.

## Materials and Methods

Between 1994 and 1998, 36 excursions to Eungella National Park were made as part of a mark–recapture study of populations of frogs associated with streams. Frogs were captured from six study sites (see Figure 1), which were visited monthly from March 1994 to June 1996, in February and September 1997, and in February and May 1998. When caught for the first time, frogs were toe-clipped using the numbering system devised by Hero (1989) and then released alive. Amputated toe tips were preserved and individually stored in vials filled with 70% ethanol or 10% formalin. To minimize effects on the animals, no further toes were taken from subsequent recaptures of marked frogs; recaptured frogs were identified and released alive.

All frog species encountered were monitored, with a special focus on T. eungellensis because of its precarious conservation status and history of decline.

In 1997, toes from six species (278 individual frogs) were histologically prepared in transverse sections stained with Ehrlich's haematoxylin for skeletochronological assessment (see Castanet et al. [1996] for a description of the technique). We subsequently reexamined these sections for B. dendrobatidis (Berger et al. 1999b; Pessier et al. 1999). On average, 160 sections per individual were examined. Despite the sections not being stained with eosin, which highlights the keratinised layer of the epidermis where B. dendrobatidis occurs (Berger et al. 1999b; Pessier et al. 1999), most diagnoses were unambiguous. A small number, however, were not, and those samples were excluded from the analysis. For this assessment, samples were labelled “positive” only when the examiners were convinced that the sample was infected with B. dendrobatidis. Samples in which no infection was found were labelled “negative.”

For this study, a further 196 archived toes of T. eungellensis were analysed by the Australian Animal Health Laboratory in Geelong, Australia, for B. dendrobatidis infection using histopathology with immunoperoxidase staining (Berger et al. 2002). The immunoperoxidase stain improves the sensitivity of diagnosis by highlighting structures that are equivocal with haematoxylin and eosin, and that would otherwise be diagnosed as negative.

With the software MARK (Cooch and White 2002), we used mark–recapture modelling to investigate the survival of T. eungellensis at different sites. Frogs were grouped into six categories on the basis of their status at first capture (infected and uninfected males, females, and subadults). For both mark–recapture and analysis of prevalence, we grouped the data into 3-mo austral seasons (in Australia, summer runs from 1 December to the end of February, etc.). Mark–recapture modelling of survival requires that several assumptions be satisfied. Most critically, every marked individual present in the population at a given time has the same probability of recapture as all other members of its group, and the same probability of surviving to the next time interval. We used a parametric bootstrap (Cooch and White 2002) to test the goodness of fit of our capture history sets to these assumptions. We analysed prevalence data using logistic models implemented in R (Version 1.6.2) (R Development Core Team 2003). Since the mark–recapture study was performed with no knowledge of the infection status of individual animals, and the examination of histology was carried out with no knowledge of details of the fate of individual animals in the field, the study was double-blinded in design.

## Abbreviations

AIC: Akaike Information Criterion
Δdev: change in deviance
se: standard error
